# Alternating processes of dry and wet nitrogen deposition have different effects on the function of canopy leaves: Implications for leaf photosynthesis

**DOI:** 10.3389/fpls.2022.1105075

**Published:** 2023-01-09

**Authors:** Chunze Wu, Siyu Zhou, Xin Cheng, Xing Wei

**Affiliations:** ^1^ School of Forestry, Northeast Forestry University, Harbin, Heilongjiang, China; ^2^ Key Laboratory of Sustainable Forest Ecosystem Management-Ministry of Education, Northeast Forestry University, Harbin, China

**Keywords:** canopy leaves, dry nitrogen deposition, wet nitrogen deposition, leaf morphology, leaf anatomy, leaf photosynthesis

## Abstract

Canopy leaves are sinks of dry and wet nitrogen (N) deposition, most studies have not considered the response of canopy leaves to the alternating processes of dry and wet N deposition. We manipulated a close top chamber experiment to observe the effects of simulated N deposition with the same total deposition flux but different dry to wet ratios on leaf structure and physiology by spraying NH_4_Cl solution or supplying gaseous NH_3_ over the canopy of seedlings of three species (*Betula platyphylla*, *Fraxinus mandshurica*, *Pinus koraiensis*) placed in the chamber. After 32 days of N deposition and relative to the control, the leaf morphology and mesophyll tissue structure of the three species had no significant changes under all N deposition treatments. With the increase in the ratio of dry to wet N deposition, the N concentration, N metabolizing enzyme activity and soluble protein concentration in leaves of all three species increased continuously, but for the leaf light-saturated net photosynthesis rate, *B. platyphylla* showed a continuous increase, *F. mandshurica* showed a continuous decrease, and *P. koraiensis* showed no significant change. We found that *F. mandshurica* was the only species whose foliar chlorophyll and potassium concentration decreased with the increase in the ratio of dry to wet N deposition and its leaf light-saturated net photosynthesis rate was positively correlated with foliar chlorophyll and potassium concentration, respectively. Our results indicate that dry deposition is relatively more important on leaf physiological functions in alternating deposition. *B. platyphylla* and *P. koraiensis* may better acclimate to canopy NH_3_/NH_4_^+^ deposition than *F. mandshurica*. Most importantly, the results indicate that a single simulated dry and wet deposition would overestimate and underestimate the response of leaf function to atmospheric N deposition, respectively. Alternating processes of dry and wet deposition should be considered for more realistic assessments of the effects of atmospheric N deposition in forests.

## 1 Introduction

Human activities have led to changes in the reactive nitrogen (Nr) component (NH_x_, reduced form; NO_y_, oxidized form) of nitrogen (N) deposition over much of the globe, NH_x_ is gradually becoming the dominant form of deposited Nr (Gu et al., 2015; Li et al., 2016; Warner et al., 2017; Liu et al., 2020a). Previous studies on the response of plant morphology and function to N deposition have been based mainly on two independent types of experiments of spraying dissolved state Nr to the soil or canopy leaves (Liu et al., 2018; Liu et al., 2020c; Li et al., 2021) and exposing plants to gaseous state Nr (Sheppard et al., 2011). However, the alternating dry and wet N deposition caused by rainfall together constitute a significant component of N deposition in natural ecosystems (Pan et al., 2012; Xu et al., 2015; Liu et al., 2020a). The contribution of dry and wet N deposition to total N deposition per unit time varies greatly between geographic regions. For example, in China, the contribution of dry N deposition to annual total N deposition ranged from 21% to 71.9% (Pan et al., 2012; Shen et al., 2013). The morphology and function of plants will be altered by both dry and wet N deposition (Adrizal et al., 2006; Sparks, 2009; Khan et al., 2020; Zhu et al., 2020), so studies that neglect the alternating processes of dry and wet N deposition may incorrectly estimate the impacts of N deposition on plant growth.

The canopy is the first tissue of plants to come into contact with dry and wet N deposition. These deposited gaseous and dissolved Nr are mainly intercepted by the canopy leaves and absorbed by ion exchange in the cuticle and/or simple diffusion in the stomata and enter the apoplast (Rennenberg and Gessler, 1999; Sparks, 2009), which are then transported to the cells and assimilated and utilized by various enzyme systems such as nitrate reductase (NR), glutamine synthetase/glutamate synthase (GS/GOGAT) cycle in chloroplasts or plastids (Krupa, 2003; Sparks, 2009). This process eventually leads to an increase in leaf N content and N metabolites, which in turn induces physiological responses and affects photosynthetic carbon assimilation capacity (Castro et al., 2008; Mao et al., 2018; Hu et al., 2019; Wu et al., 2022), and also causes changes in leaf morphological and anatomical traits (Zhu et al., 2020; Khan et al., 2020). Therefore, canopy leaves are also the first vegetative organ of plants to respond to dry and wet N deposition.

However, in the results of previous gaseous and dissolved Nr addition studies, plants exposed to certain concentrations of gaseous Nr generally had higher N concentrations or recovery in leaves and shoots than when dissolved Nr was applied to the root medium or canopy. (Pérez-Soba and van der Eerden, 1993; Castro et al., 2008; Wang et al., 2021). For example, Jones et al. (2008) supplied ^15^NH_3_ to *Calluna vulgaris* in the flux chamber and found that 22% of applied NH_3_ was retained by leaf stomata and 53% by green shoots and leaf cuticles. Adriaenssens et al. (2012) sprayed four temperate tree species with ^15^NH_4_^+^ and ^15^NO_3_^-^ solutions and found that only 1-3% of the applied N was retained by foliage and twigs. This difference indicated that plants may respond more rapidly and to a greater extent to dry N deposition than to wet N deposition, for example, Sheppard et al. (2011) found that dry deposition of gaseous NH_3_ drives cover of ombrotrophic bog species change faster than equivalent doses of wet deposition of NH_4_Cl solution.

Although previous studies conducted by applying gaseous or dissolved Nr to the canopies have improved our understanding of the retention, assimilation of Nr in plant canopy leaves, however, applying a single state of Nr is not representative of the N deposition in natural ecosystems, and the response of plants to it may also not accurately assess the effects of atmospheric N deposition on plants or ecosystems. Our understanding of the response of photosynthesis and photosynthetic system components in canopy leaves to the alternating processes of dry and wet N deposition and leaf sensitivity/tolerance to different states of Nr remains incomplete. Therefore, under the change of composition and state of atmospheric N deposition (Li et al., 2016; Warner et al., 2017; Liu et al., 2020a), revealing the changes in leaves morphological, anatomical and physiological traits under the alternating processes of dry and wet NH_x_ deposition is critical for a more comprehensive understanding and accurate assessment of the effects of N deposition on plants and even forest ecosystem.

For many tree species, NH_4_^+^ toxicity will occur in the condition of high NH_4_^+^-level root medium, and trees usually show different NH_4_^+^ sensitivity/tolerance in response to increased NH_4_^+^ levels in the root medium due to different levels of preference for NH_4_^+^ (Cui and Song, 2007; Esteban et al., 2016). This sensitivity depends on tree species and their life forms and growth character (Cui and Song, 2007; Esteban et al., 2016). In this study, we conducted a short-term close top chamber experiment to simulate NH_x_ deposition with different dry to wet ratios over the canopy of seedlings of three species: *Betula platyphylla* (lower NH_4_^+^ sensitivity) (Crabtree and Bazzaz, 1993; Zhang and Cui, 2011), *Fraxinus mandshurica* (higher NH_4_^+^ sensitivity) (Zhang et al., 2000; Wu et al., 2003), *Pinus koraiensis* (lower NH_4_^+^ sensitivity) (Cui and Song, 2007). Our aim was to more comprehensively understand the effects of atmospheric N deposition on plant canopy leaves by comparing the leaf morphology, anatomical structure and physiology responses of different NH_4_^+^-sensitive seedlings under simulated NH_x_ deposition. We hypothesized that: (1) at the same total N deposition flux, dry deposition would have a greater effect on the morphology, anatomy and physiology of plant leaves than wet deposition; (2) the sensitivity of trees to canopy deposited NH_x_ and to NH_4_^+^ in the root medium would be similar; this hypothesis was developed based on the findings of some previous studies that plants also show different sensitivity/tolerance in response to increased environmental NH_3_ concentration (Pitcairn et al., 1998; Adrizal et al., 2006).

## 2 Materials and methods

### 2.1 Plant material

In May 2021, *B. platyphylla* clones obtained from tissue culture were prepared in nutrient pots in a greenhouse, and then transferred to new nutrient pots (10×10×10 cm) together with one-year-old *F. mandshurica* seedlings and two-year-old *P. koraiensis* seedlings in late June. The source of culture substrate remained the same, which were 1:1 (v: v) black soil-vermiculite, black soil, 1:1 black soil-vermiculite respectively. In early July, 72 relatively uniform seedlings of each species were selected according to their height and basal diameter and transferred to the chamber for one week before starting the experiment. Each chamber contains three species, with 12 seedlings per species. The characteristics of the plant material and soil are shown in **Table 1**.

**Table 1:** Characterization of plant material and soil properties in chambers.

	plant properties					soil properties		
species	Life forms	Leaf types	Growth forms	Growth character	Seedling height (cm)	Total C (%)	Total N(%)	pH
*Betula platyphylla*	deciduous	broad	shade-intolerant	fast-growing	37.6	10.42	0.61	5.1
*Fraxinus mandshurica*	deciduous	broad	shade-tolerant	slow-growing	13.3	9.96	0.67	4.8
*Pinus koraiensis*	evergreen	needle	shade-tolerant	slow-growing	14.2	11.06	0.89	5.5

### 2.2 Experimental design

The close top chamber experiment was performed in the greenhouse of Northeast Forestry University in Harbin, China. The temperature in the chamber during the experiment was 25-32°C, the relative humidity was 55-60%, and the maximum illuminance was 23,600lux. We used a split-plot design, the main plots are six N application treatments conducted within six chambers respectively, three replicates of each treatment. N application treatments included a control that applied activated carbon filtered ambient air and deionized water, and five types of N deposition with the same total deposition flux but different dry to wet ratios simulated on the basis of control (0D+100W, the ratio of dry to wet N deposition (*R*_Dry/Wet_) was 0:100; 25D+75W, *R*_Dry/Wet_ was 25:75; 50D+50W, *R*_Dry/Wet_ was 50:50; 75D+25W, *R*_Dry/Wet_ was 75:25; 100D+0W, *R*_Dry/Wet_ was 100:0). Three species were assigned to each split plots by complete block design.

The simulated alternating processes of dry and wet N deposition are composed of multiple 4-day dry and wet deposition cycles. One cycle of the 0D+100W treatment is composed of four consecutive days of wet deposition, one cycle of the 25D+75W treatment is composed of one day of dry deposition and three consecutive days of wet deposition, one cycle of the 50D+50W treatment is composed of two consecutive days of dry deposition and two consecutive days of wet deposition, one cycle of the 75D+25W treatment is composed of three day of dry deposition and one day of wet deposition and one cycle of the 100D+0W treatment is composed of four consecutive days of dry deposition.

**Figure 1:**
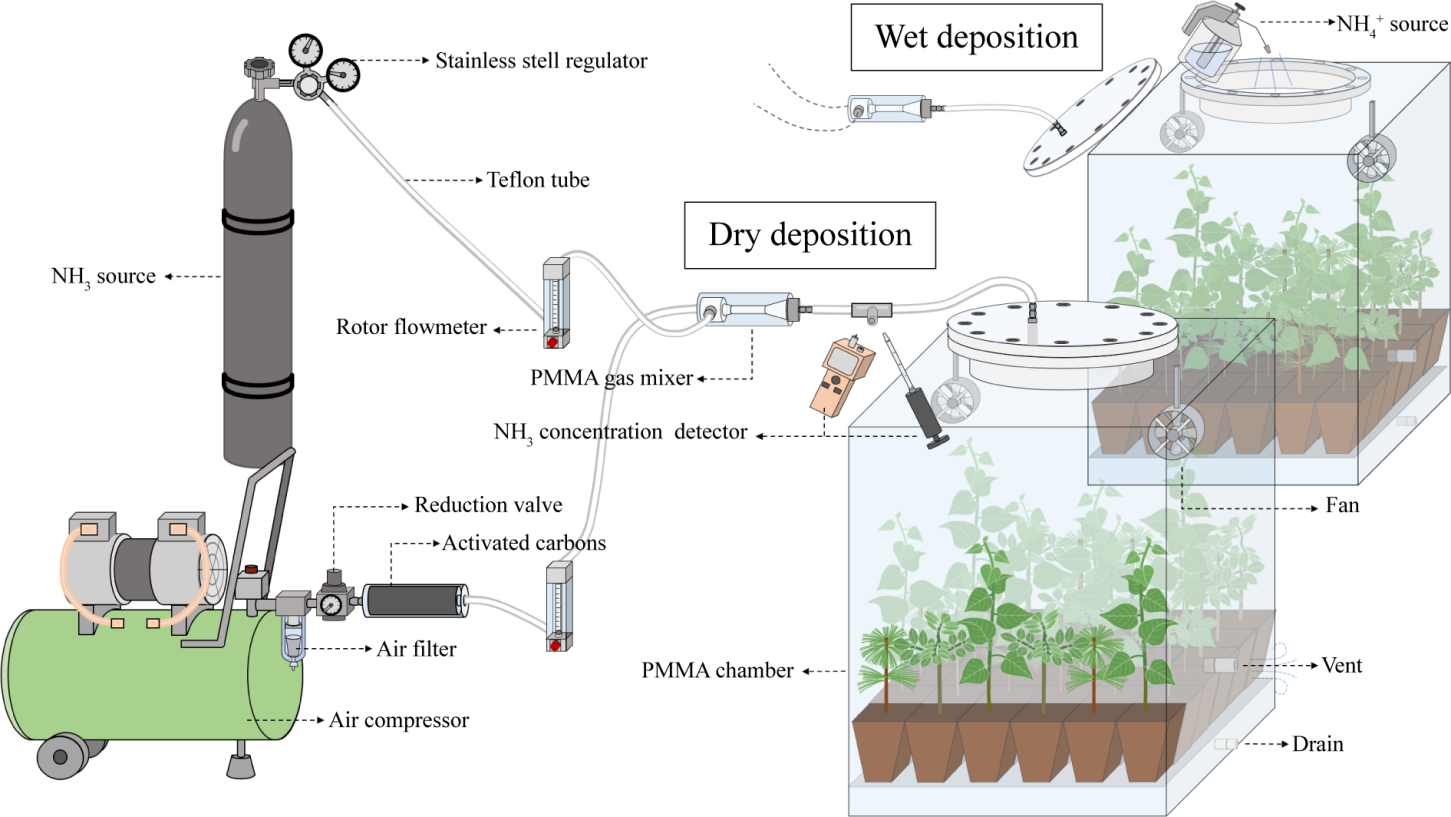
A schematic diagram showing the materials used to simulating dry and wet N deposition The ambient air compressed by the air compressor is pumped into the chamber from the top of the chamber after passing through the reduction valve, activated carbons, rotor flowmeter and gas mixer in turn. NH_3_ is injected into the gas mixer from a cylinder and mixed with the pumped air, and adjusted to the desired concentration by rotor flowmeter to simulate dry deposition. The top of the chamber consists of a flange interface that can be opened and used to simulate wet deposition. PMMA, polymethyl methacrylate.

The dry deposition was simulated by injecting gaseous NH_3_ at a concentration of 330 ± 10 μg m^-3^ of N into the chamber for 14 hours (5:00 h-19:00 h) per day. The ambient air compressed by the air compressor is pumped into the chamber (0.6×0.6×0.7 m) from the top of the chamber after passing through the reduction valve, activated carbons (to remove NH_3_ from the ambient air), rotor flowmeter and gas mixer in turn, the flow rate is 30 L min^-1^. NH_3_ is injected into the gas mixer from a cylinder containing with a calibrated gas mixture of 0.1% NH_3_ in N_2_ and mixed with the pumped air, adjusted to the desired concentration by rotor flowmeter. All tubes in contact with NH_3_ are made of Teflon, fans are installed in the chamber to reduce the difference in NH_3_ concentration over horizontal space. NH_3_ concentration in the inlet and outlet of the chamber is measured by pump suction NH_3_ detector (JA908-NH_3_; Yongqi Co. Ltd, GuangDong, China) and NH_3_ detection tube (3CG Ammonia; GASTEC, Tokyo, Japan). The gas exchange rate in the chamber was 30 L min^-1^ and the concentration of NH_3_ emitted from each chamber vent during the simulated dry deposition is less than 28 μg m^-3^ of N. After correcting the amount of NH_3_ adsorption by the chamber inner wall, the dry N deposition flux was calculated from the difference of NH_3_ concentration between the inlet and outlet of the chamber. The daily dry deposition flux was 7.06 ± 0.25 mg of N (see **Figure 1**).The wet deposition was simulated by uniformly spraying 305 ml of NH_4_Cl solution at a concentration of 23.1 mg L^-1^ of N over the canopy at 05:00 h per day. The daily wet deposition flux was 7.05 mg of N (see **Figure 1**). The total deposition flux for each N application treatment was equivalent to 72 kg ha^-1^ yr^-1^ of N, which was close to the high N level in the range of N deposition rates (0-70 kg ha^-1^ yr^-1^ of N) in different geographical regions of China (Wen et al., 2020).

Each N deposition treatment had the same gas exchange rate and the amount of deionized water applied. The same amount of water (deionized water, 305 ml) as in the simulated wet deposition was applied daily in the control and simulated dry deposition. To avoid additional adsorption of NH_3_ by the water film formed on the leaves after canopy spraying water (which is not representative of true dry N deposition process), the deionized water in the dry deposition treatment was applied to the soil surface through a syringe.

### 2.3 Leaf photosynthesis and sample collection

Between 09:00 h and 11:30 h on days 17 and 33 after the start of the N application, leaf light-saturated net photosynthesis rate (Pmax) was measured *in situ* using a LI-6400 with an integrated LED light source (Li-Cor, Lincoln, NE, USA). The chamber CO_2_ concentration was maintained at ambient level. To obtain light-saturated rates of photosynthesis, the leaf in the chamber was illuminated at 1000 μmol m^-2^ s^-1^ photosynthetic photon flux density. According to preliminary trials, we found that the CO_2_ uptake of all three species light saturated at 1000 μmol photons m^-2^ s^-1^. For each measurement, we randomly selected five seedlings from each species in per treatment. We selected mature, healthy, and integrated leaves located at the 3rd and 4th position from the top of *B. platyphylla*, the 1st and 2nd position from the top of compound leaves of *F. mandshurica*, and two clusters of current-year needles of *P. koraiensis* to measure Pmax. Recalibrate the Pmax of the needle after measuring the projected area of the needles. Averaged values of these two leaves (two clusters of needles) were used to represent the response of this seedling.

After the second photosynthesis measurements, leaf sampling was performed. The leaves that had been used for photosynthesis measurements were collected, part of the sample was wrapped in tinfoil and stored in liquid nitrogen, while the other part of the sample was put into a sealed plastic bag and placed in an ice box. we then randomly selected five seedlings from each species in per treatment, leaves located at the 3rd to 7th position from the top of *B. platyphylla*, the 2nd-6th position from the top of compound leaves of *F. mandshurica* and the current-year needles of *P. koraiensis* were collected. All samples collected were mature, healthy and integrated. Two leaves (two clusters of needles) from each seedling were gently washed in deionized water and immediately chemically fixed in Formalin-Aceto-Alcohol (FAA) solution (70% ethanol: glycerine: glacial acetic acid = 18:1:1) separately, the rest of the leaves were put into separate sealed plastic bag and placed in an ice box. All fresh samples were transported to the laboratory for immediate processing or analysis, samples stored in liquid nitrogen were transported to the laboratory and stored at -80°C and fixed samples were stored at 4°C, until further analysis.

### 2.4 Leaf morphology and foliar chemistry

The fresh leaf samples that were not used for photosynthesis measurements were washed with deionized water and fresh weight was measured. The leaf samples are scanned by a color scanner (12000XL; Expression, Nagano, Japan), and the scanned images were analyzed with root system analyzer software (WinRHIZO Pro 2016; Regent instruments Co., Ltd, Québec, Canada) to determine leaf area and projected area of needles (LA). The leaf samples were then oven-dried at 65°C until constant weight then weighed. Leaf mass per area (LMA) and specific leaf area (SLA) were calculated. Dried leaf samples were ground into fine powder using a ball mill (MM400; Retsch, Haan, Germany). The N concentration was determined using a macro elemental analyzer (vario MACRO CN; Elementar, Langenselbold, Germany). To measure the concentration of phosphorus (P) and potassium (K) in leaves, the fine powder was digested with sulphuric and perchloric acid. The P concentration was determined using a continuous flow analyzer (Auto Analyzer 3; SEAL Analytical Co., Ltd, Norderstedt, Germany), based on the ascorbic acid molybdate analysis, and the K concentration was determined using a flame photometer.

### 2.5 Foliar soluble proteins and photosynthetic pigments

For photosynthetic pigments analysis, fresh leaves that had been used for photosynthesis measurements were shredded after removing the major vein, 0.1g of shredded leaf tissue was placed in 10 ml of 80% acetone in the dark for 24 h, and centrifuged at 10,000*g* for 10 min. The supernatant was scanned at *λ*_663_, *λ*_645_ and *λ*_470_ (TU-1950; Presee Co., Ltd, Beijing, China) and the concentrations of chlorophylls (chlorophyll/Chl*a*, Chl*b*) and carotenoids were quantified as per Zhang et al. (2020). For soluble protein analysis, leaves stored at -80°C were shredded after removing the major veins, 0.2 g of shredded leaf tissue were homogenized in 5ml deionized water, and then centrifuged at 10,000*g* for 30 min, and the supernatant was used for protein analysis according to the Kormas Brilliant Blue method.

### 2.6 Foliar nitrogen-metabolizing enzymes

The activities of nitrate reductase (NR, EC 1.6.6.1), glutamine synthetase (GS, EC 6.3.1.2) and glutamate synthase (Fd-GOGAT EC 1.4.7.1) in leaves stored at -80°C were measured using enzyme-linked immunosorbent assay (ELISA). Solid-phase antibody was made using purified plant NR (or GS, GOGAT) antibody. Then, combined with antibody labelled with horseradish peroxidase (HRP), NR (or GS, Fd-GOGAT) was added to microtiter plate wells to become an antibody-antigen-enzyme-antibody complex. This complex became blue with 3,3′,5,5′-tetramethyl benzidine (TMB) substrate solution after complete washing and converted to final yellow under the action of acid. The optical density (OD) values were measured spectrophotometrically at a wavelength of 450 nm to compare with the standard curves to determine the activity of NR (or GS, Fd-GOGAT) in the samples.

### 2.7 Leaf tissue anatomy

Leaf anatomical traits were determined following the protocols described by Guo et al. (2008). Leaves of broadleaved species fixed in the FAA were cut into small pieces of 1×2 cm along the main veins in the middle part of the leaves, and fixed needles were cut into small segments of 0.5 cm in length in their middle part. This excised leaf tissue was embedded in paraffin individually after dehydration by immersion in a sequence of alcohol solutions and cut into sections of 8μm thickness and then stained with safranin-fast green. The slides were photographed under a compound microscope (BX51; Olympus, Tokyo, Japan). The abaxial (ABE) and adaxial (ADE) epidermis thickness, spongy mesophyll thickness (SMT), palisade mesophyll thickness (PMT) and leaf thickness of broadleaved species, the combined epi-hypodermis thickness (EHT) and mesophyll area of *P. koraiensis* were extracted from the images using Motic Images Advanced 3.2.

### 2.8 Statistical analyses

The effect of N deposition on various traits of leaf morphology, anatomical structure, and physiology were assessed using one-way ANOVA, and least significant difference tests were conducted to compare means of these trait variables among treatments. Linear regression analyses were used to determine the relationships between Pmax and foliar K concentration, foliar chlorophyll concentration. All variables were tested for the normality of their distributions. Nonnormally distributed variables were log-transformed to meet normality assumptions before performing ANOVA. All statistical analysis mentioned above was performed using the SPSS software v19.0 (SPSS Inc., Chicago, IL, USA).

## 3 Results

### 3.1 Foliar chemistry

**Figure 2:**
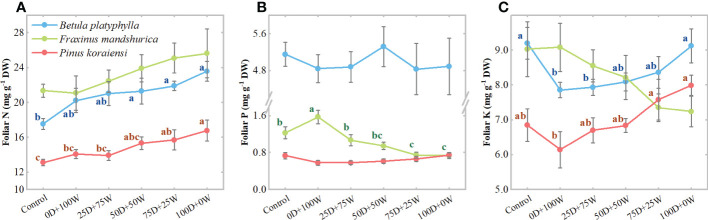
Concentrations (mg g^-1^ DW, **A–C**) of foliar: nitrogen (N), phosphorus (P) and potassium (K) of three species affected by six canopy N application treatments for 32 days. N application treatments included a control (no application of N) and five types of N deposition with dry to wet ratios of 0:100 (0D+100W), 25:75 (25D+75W), 50:50 (50D+50W), 75:25 (75D+25W) and 100:0 (100D+0W), respectively. Error bars indicate the standard error of the mean (*n* = 5). Within the same species in each panel, different letters on the left side of the error bar demonstrate significant differences among six treatments (*P* < 0.05) according to the least significant difference test. The scales of the *y*-axis are different in each panel.

Under N application treatments, the foliar N concentrations of the three species increased continually with the increase in *R*_Dry/Wet_ (**Figure 2A**). Relative to the control, the foliar N concentrations of the three species under the 0D+100W treatment did not show significant differences, while the foliar N concentrations of *B. platyphylla*, *F. mandshurica* and *P. koraiensis* under the 100D+0W treatment increased by 16.6% (*P* = 0.051), 21.5% (*P* = 0.101) and 19.2% (*P* < 0.01), respectively. The foliar P concentrations of *B. platyphylla* and *P. koraiensis* did not show significant differences among all treatments, only the foliar P concentration of *F. mandshurica* decreased continually with the increase in *R*_Dry/Wet_ (**Figure 2B**). Relative to the control, the foliar K concentrations of *B. platyphylla* and *P. koraiensis* under the 0D+100W treatment decreased by 14.6% (*P* = 0.017) and 10.3% (*P* = 0.254), respectively, but increased continually with the increase in *R*_Dry/Wet_. The foliar K concentration of both *B. platyphylla* and *P. koraiensis* under the 100D+0W treatment was not significantly different compared with that under the control (**Figure 2C**). It is noteworthy that the foliar K concentration of *F. mandshurica* did not show significant differences among all treatments but showed a continuous decrease with the increase in *R*_Dry/Wet_. Relative to the control, the foliar K concentration of *F. mandshurica* under the 100D+0W treatment decreased by 19.8% (*P* = 0.041) (**Figure 2C**).

### 3.2 Nitrogen -metabolizing enzymes activities

**Figure 3:**
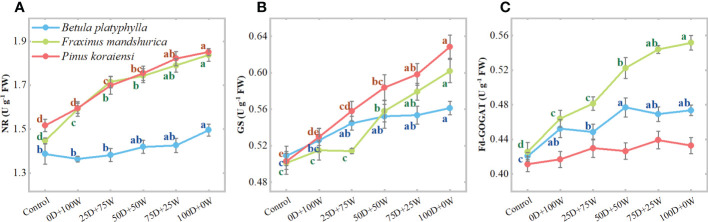
Activities (U g^-1^ FW, **A-C**) of nitrate reductase (NR), glutamine synthetase (GS) and glutamate synthase (Fd-GOGAT) in leaves of three species affected by six canopy nitrogen (N) application treatments for 32 days. N application treatments included a control (no application of N) and five types of N deposition with dry to wet ratios of 0:100 (0D+100W), 25:75 (25D+75W), 50:50 (50D+50W), 75:25 (75D+25W) and 100:0 (100D+0W), respectively. Error bars indicate the standard error of the mean (*n* = 5). Within the same species in each panel, different letters on the left side of the error bar demonstrate significant differences among six treatments (*P* < 0.05) according to the least significant difference test. The scales of the *y*-axis are different in each panel.

Relative to the control, except for the reduced NR activity in *B. platyphylla* leaves under the 0D+100W and 25D+75W treatments, the activities of NR, GS, and GOGAT in leaves of all three species increased under each N application treatment and increased continually with the increase in *R*_Dry/Wet_ (**Figures 3A–C**). Compared with the 0D+100W treatment, the NR activities in *B. platyphylla* leaves, *F. mandshurica* leaves and *P. koraiensis* needles under the 100D+0W treatment increased by 9.0% (*P* < 0.01), 14.5% (*P* < 0.01) and 15.1% (*P* < 0.01), respectively; the GS activities in *B. platyphylla* leaves, *F. mandshurica* leaves and *P. koraiensis* needles under the 100D+0W treatment increased by 6.7% (*P* = 0.026), 16.9% (*P* < 0.01), and 18.7% (*P* < 0.01), respectively; the Fd-GOGAT activities in *B. platyphylla* leaves, *F. mandshurica* leaves and *P. koraiensis* needles under the 100D+0W treatment increased by 4.7% (*P* = 0.093), 18.9% (*P* < 0.01), and 3.8% (*P* = 0.249), respectively.

### 3.3 Soluble proteins and photosynthetic pigments concentrations

**Figure 4:**
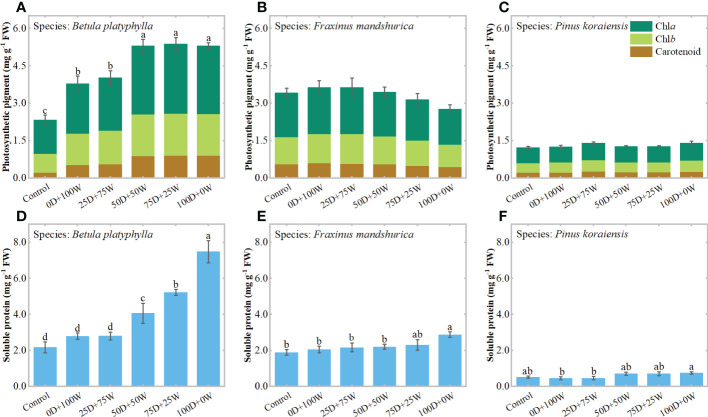
Concentrations of photosynthetic pigment (mg g^-1^ FW, **A–C**) and soluble protein (mg g^-1^ FW, **D-F**) in leaves of three species affected by six canopy nitrogen (N) application treatments for 32 days. N application treatments included a control (no application of N) and five types of N deposition with dry to wet ratios of 0:100 (0D+100W), 25:75 (25D+75W), 50:50 (50D+50W), 75:25 (75D+25W) and 100:0 (100D+0W), respectively. Error bars indicate the standard error of the mean of chlorophyll (*a* + *b*) concentration and soluble protein concentration (*n* = 5). Different letters above the error bar represent significant differences among six treatments (*P* < 0.05) according to the least significant difference test.

Relative to the control, the chlorophyll concentration in *B. platyphylla* leaves increased significantly under each N application treatment and increased continually with the increase in *R*_Dry/Wet_ (**Figure 4A**), which increased by 54.9% (*P* < 0.01) under the 0D+100W treatment and increased by 108.1% (*P* < 0.01) under the 100D+0W treatment. The chlorophyll concentrations in *F. mandshurica* leaves and *P. koraiensis* needles did not show significant differences among all treatments, it is noteworthy that the chlorophyll concentration in *F. mandshurica* leaves showed a continuous decrease with the increase in *R*_Dry/Wet_ (**Figures 4B, C**). Relative to the control, the chlorophyll concentration in *F. mandshurica* leaves increased by 5.9% (*P* = 0.64) under the 0D+100W treatment and decreased by 19.4% (*P* = 0.134) under the 100D+0W treatment.

Relative to the control, the soluble protein concentrations in *B. platyphylla* leaves, *F. mandshurica* leaves and *P. koraiensis* needles under the 0D+100W treatment did not show significant differences but increased continually with the increase in *R*_Dry/Wet_, the soluble protein concentrations in their leaves increased by 245.0% (*P* < 0.01), 52.3% (*P* < 0.01) and 45.3% (*P* = 0.063) under the100D+0W treatment, respectively (**Figures 4D–F**).

### 3.4 Leaf photosynthesis rates

**Figure 5:**
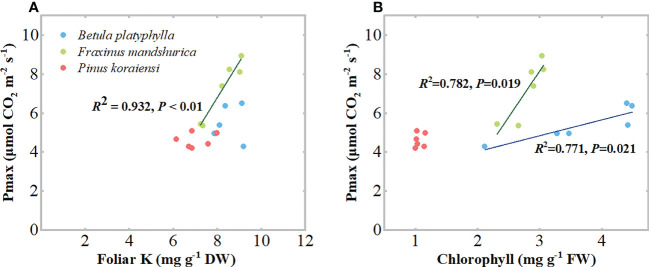
Leaf light-saturated net photosynthesis rate (Pmax, μmol CO_2_ m^-2^ s^-1^) of three species affected by six canopy nitrogen (N) application treatments for 16 **(A)** and 32 **(B)** days. N application treatments included a control (no application of N) and five types of N deposition with dry to wet ratios of 0:100 (0D+100W), 25:75 (25D+75W), 50:50 (50D+50W), 75:25 (75D+25W) and 100:0 (100D+0W), respectively. Error bars indicate the standard error of the mean (*n* = 5). Within the same species in each panel, different letters on the left side of the error bar demonstrate significant differences among six treatments (*P* < 0.05) according to the least significant difference test.

After 16 and 32 days of N application and relative to the control, the Pmax of *B. platyphylla* under the 0D+100W treatment increased by 7.0% (*P* = 0.382) and 15.2% (*P* = 0.385), respectively. For *F. mandshurica*, the Pmax under the 0D+100W treatment increased by 4.3% (*P* = 0.675) and 10.2% (*P* = 0.197), respectively. However, with the increase in *R*_Dry/Wet_, the Pmax of *B. platyphylla* on both measurement days increased continually, while that of *F. mandshurica* on both measurement days decreased continually. Relative to the control, the Pmax of *B. platyphylla* under the 100D+0W treatment increased by 28.3% (*P* < 0.01) and 51.3% (*P* < 0.01) after 16 and 32 days of N application, respectively. For *F. mandshurica*, the Pmax under the 100D+0W treatment decreased by 44.6% (*P* < 0.01) and 32.9% (*P* < 0.01) on two measurement days, respectively. Moreover, after 32 days of N application, there were significantly positive relationships between Pmax and foliar chlorophyll concentrations in *B. platyphylla* and *F. mandshurica*, for *F. mandshurica*, Pmax also had a positive relationship with foliar K concentration (**Figures 5A, B**). There was no significant difference in Pmax of *P. koraiensis* among all treatments on two measurement days (**Figures 5A, B**).

### 3.5 Leaf morphology and anatomy

The leaf area, SLA and LMA of the three species did not show significant differences among all treatments (**Table S1**), but some of the leaf anatomical traits were significantly affected by N application. Relative to the control, the ADE of *B. platyphylla* and *F. mandshurica* under the 0D+100W treatment increased by 66.0% (*P* < 0.01) and 23.2% (*P* < 0.01), respectively, but decreased continually with the increase in *R*_Dry/Wet_ (**Table 2**; **Figure S2**). The ADE of *B. platyphylla* and *F. mandshurica* under the 100D+0W treatment was not significantly different compared with that under the control. Relative to the control, the EHT of *P. koraiensis* increased under each N application treatment and showed significance only under the 50D+50W treatment. Besides, the ABE and leaf thickness of *B. platyphylla* showed the same trend as ADE under the N application treatments but were less affected by the N application.

**Table 2:** Leaf anatomical traits of three species affected by six canopy nitrogen (N) application treatments for 32 days.

Treatment						
Species/anatomical traits	Control	0D+100W	25D+75W	50D+50W	75D+25W	100D+0W
*Betula platyphylla*						
ADE (μm)	8.89 ± 0.76b	14.75 ± 0.94a	14.65 ± 1.31a	10.59 ± 0.63b	9.77 ± 0.67b	9.37 ± 0.84b
ABE (μm)	8.19 ± 0.72ab	9.53 ± 0.60a	8.67 ± 0.57ab	8.09 ± 0.41b	7.76 ± 0.30b	7.72 ± 0.19b
PMT (μm)	22.80 ± 1.01	23.25 ± 0.71	22.86 ± 1.11	23.01 ± 0.58	21.02 ± 1.12	21.36 ± 0.48
SMT (μm)	36.28 ± 3.64	36.45 ± 2.11	36.71 ± 2.95	35.23 ± 1.38	33.14 ± 1.99	30.08 ± 1.56
Leaf thickness (μm)	77.35 ± 5.89ab	83.45 ± 4.14a	82.58 ± 4.30a	77.31 ± 2.59ab	69.67 ± 3.49b	68.50 ± 2.17b
*Fraxinus mandshurica*						
ADE (μm)	16.07 ± 0.52a	19.80 ± 1.19ab	17.40 ± 1.14b	16.12 ± 0.96b	15.38 ± 0.49b	14.86 ± 0.56b
ABE (μm)	12.75 ± 0.60	13.44 ± 0.78	13.40 ± 0.75	13.64 ± 0.38	13.21 ± 0.24	13.44 ± 0.43
PMT (μm)	69.21 ± 4.02	69.11 ± 1.84	66.04 ± 6.11	65.00 ± 1.66	64.61 ± 1.79	63.67 ± 2.42
SMT (μm)	113.82 ± 10.79	110.13 ± 4.49	108.92 ± 7.21	104.06 ± 4.42	105.56 ± 4.46	105.96 ± 6.69
Leaf thickness (μm)	215.39 ± 15.94	215.38 ± 6.18	206.19 ± 14.26	199.09 ± 6.08	198.20 ± 5.76	200.04 ± 9.31
*Pinus koraiensis*						
EHT (μm)	20.78 ± 0.49b	21.49 ± 0.31ab	22.11 ± 0.63ab	22.47 ± 0.47a	21.57 ± 0.31ab	21.62 ± 0.26ab
Mesophyll area (mm^2^)	0.3708 ± 0.0302	0.3997 ± 0.0245	0.3728 ± 0.0325	0.3569 ± 0.0277	0.3415 ± 0.0098	0.3414 ± 0.0133

ADE, adaxial epidermis thickness; ABE abaxial epidermis thickness; PMT, palisade mesophyll thickness; SMT, spongy mesophyll thickness; EHT, combined epi-hypodermis thickness. N application treatments included a control (no application of N) and five types of N deposition with dry to wet ratios of 0:100 (0D+100W), 25:75 (25D+75W), 50:50 (50D+50W), 75:25 (75D+25W) and 100:0 (100D+0W), respectively. Values are mean ± 1 SE (n = 5). Different letters after means in each row denote significant differences among treatments (P < 0.05) according to the least significant difference test.

## 4 Discussion

### 4.1 The importance of considering the alternation of dry and wet nitrogen deposition

In the present study, N concentrations, GS and Fd-GOGAT activities in leaves of all three species slightly increased under wet deposition, but increased continually with the increase in *R*_Dry/Wet_ (**Figures 2A**, **3B, C**). These results confirm that atmospherically deposited NH_3_ and NH_4_^+^ can be used as a foliar supply of N (Jones et al., 2008; Wang et al., 2021) and suggest that canopy leaves may retain more NH_3_ from dry deposition compared to NH_4_^+^ from wet deposition. Leaf NH_3_/NH_4_^+^ uptake kinetics were not investigated in the present study, as the focus of the work was to understand how alternating dry and wet N deposition with the same deposition flux affects the structure and physiology of canopy leaves. In fact, the retention and assimilation of gaseous NH_3_ or dissolved NH_4_^+^ by leaves has been widely studied (Krupa, 2003; Sparks, 2009). Therefore, the fact that N concentration and N metabolic enzyme activities increased with the increase in *R*_Dry/Wet_ indicates that dry and wet deposition have different effects on foliar N status. Relative to wet deposition, dry deposition of N will lead to higher foliar N concentration.

In previous studies, leaf morphology and anatomy showed high plasticity in response to changes of leaf N levels (Khan et al., 2020; Zhu et al., 2020). However, in the present study, except for the gradient changes of epidermis (epi-hypodermis) thickness under N application, we did not find significant effects of continuously increasing N concentration on leaf morphological (i.e., the leaf area, SLA and LMA of the three species) and anatomical traits (i.e., the PMT and SMT of *B. platyphylla* and *F. mandshurica* and mesophyll area of *P. koraiensis*) of the three species (**Table 2**; **Table S1**). In our results (**Table 2**; **Figure S2**), with the decrease in *R*_Dry/Wet_, the continuous thickening of ADE in broadleaved species (*B. platyphylla* and *F. mandshurica*) may be a defense strategy for the leaves in response to the weak acidity of NH_4_Cl solution (Kateivas et al., 2022). For coniferous species (*P. koraiensis*), the EHT increased at first and then decreased (**Table 2**; **Figure S2**), suggesting that the needles may have the same defense strategy, but due to greater retention of wet deposition by needles, the EHT may be injured by higher doses of NH_4_Cl and then thinned. Although the above results were inconsistent with our first hypothesis, they were not unexpected, because the leaf morphological and structural traits may be invariable under short-term N application (Makoto and Koike, 2007).

**Figure 6:**
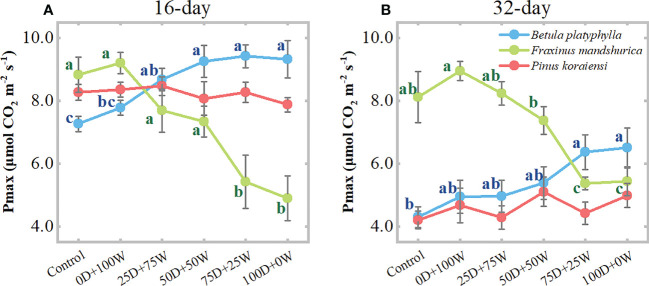
Relationships between light-saturated net photosynthesis rate (Pmax) and foliar Potassium (K) concentrations **(A)**, chlorophyll concentrations **(B)** in the three species. Within the same species, each symbol represents data from one N application treatment. N application treatments included a control (no application of N) and canopy N deposition with dry to wet ratios of 0:100, 25:75, 50:50, 75:25 and 100:0, respectively. Data were collected after 32 days of N application treatments.

We found that the continuous increase in foliar N concentrations of the three species was accompanied by a continuous increase in soluble protein concentrations in their leaves (**Figures 4D–F**; **Figure S1**). These results were consistent with previous studies that leaves can store the applied N in the form of some organic compounds in the organic N pool, so an increase in N concentration in leaves is usually accompanied by an increase in one and more amino acids, total free amino acids and proteins (Bubier et al., 2011; Hu et al., 2019). N is an important component of chlorophylls, photosynthesis-related enzymes and proteins, the addition of N inevitably changes the N levels and N metabolism in plants while also affecting the photosynthetic system composition and photosynthetic carbon assimilation capacity of plants (Tomaszewski and Sievering, 2007; Liu et al., 2020b). In the present study, the foliar chlorophyll concentrations and Pmax of *B. platyphylla* and *F. mandshurica* also showed a gradient change with the continuous increase in their foliar N concentrations. Relative to the control, the variation of these traits under dry deposition was much larger than those under wet deposition (**Figures 4A, B**, **6A, B**). The above results suggest that dry deposition has a greater effect on plant leaf physiology than wet deposition.

Although we should be careful to deduct long-term leaf functional modifications by simulated N deposition from short-term acclimation studies, our leaf physiology results in the short term already suggest that a single simulated dry and wet N deposition would overestimate and underestimate effects of atmospheric N deposition on leaf function, respectively. Therefore, alternating processes of dry and wet N deposition should not be neglected.

### 4.2 Response of leaf photosynthetic capacity of different tree species to canopy NH_x_ deposition

With the continuous increase in foliar N and soluble protein concentrations of the three species, their Pmax showed different trends. For broadleaved species, the Pmax of fast-growing *B. platyphylla* increased continuously, while the Pmax of slow-growing *F. mandshurica* decreased continuously (**Figures 6A, B**), and there was a significant positive correlation between Pmax and foliar chlorophyll concentrations for both species (**Figure 5B**). These results indicate that some of the excess N can be invested in light harvesting and photosynthesis in *B. platyphylla* leaves, while N was diverted away from the photosynthetic system of *F. mandshurica* leaves and photosynthetic capacity was suppressed. The divergent response of photosynthesis between *B. platyphylla* and *F. mandshurica* seems to be expected, because slow-growing species may lack a better strategy to use excess N input into their leaves than fast-growing species (Aerts and Chapin, 1999; Li et al., 2012; Mikola et al., 2021). For coniferous species, there was no significant change in Pmax and foliar chlorophyll concentration of *P. koraiensis* (**Figures 4C**, **6A, B**). These results were comparable to the response of needles under high levels of N addition in previous studies (Warren et al., 2003; Bauer et al., 2004; Li et al., 2022), which suggested that the excess N in needles is stored as amino acids and soluble protein, rather than being invested in photosynthesis. This storage mechanism in needles is supposed to be a peculiar adaptation of evergreen species when N uptake from the environment exceeds immediate growth requirements (Warren et al., 2003; Makoto and Koike, 2007). In general, the life forms and growth character of tree species probably contribute to different results of photosynthesis among tree species under NH_x_ deposition.

In fact, there is ample evidence from previous studies that appropriate NH_4_^+^ addition can increase the investment of N in photosynthetic system components, induce an increase in photosynthetic capacity of leaves and promote plant growth (Pérez-Soba and van der Eerden, 1993; Elvir et al., 2006; Hong et al., 2021). But above a certain threshold, NH_4_^+^ will be a stress and lead to a decrease in cation (K^+^, Ca^2+^ and Mg^2+^) concentrations in plant tissues (i.e. nutrient limitation of the plant), and have a negative impact on the leaf physiological function, induce a decrease in photosynthetic capacity (Esteban et al., 2016; Mao et al., 2018; Hong et al., 2021). In the root medium, this decrease is caused by the cation leaching due to NH_4_^+^-induced soil acidification and the competition of excess NH_4_^+^ with cations at the root cell membrane level (Britto and Kronzucker, 2002; Lu et al., 2014; Esteban et al., 2016). Therefore, in the traditional view, the NH_4_^+^ tolerance threshold (i.e., sensitivity to NH_4_^+^) of tree species and the nutrient limitation mechanism when NH_4_^+^ exceeds the threshold seem to better explain the results of photosynthesis the three species in this study. However, the process of our simulated alternating NH_x_ deposition mainly occurred in the canopy and considering that the change in photosynthesis may also be related to the effect of NH_3_/NH_4_^+^ retention in leaves, the sensitivity of species to deposited NH_x_ should be revisited.

Our Pmax results showed a higher sensitivity of *F. mandshurica* to canopy NH_x_ deposition than *B. platyphylla* and *P. koraiensis*, which was consistent with our second hypothesis that the sensitivity of trees to canopy deposited NH_x_ and to NH_4_^+^ in the root medium would be similar. This hypothesis was further supported by our foliar K concentrations results, where the decrease in Pmax of *F. mandshurica* was also accompanied by a decrease in its foliar K concentration (**Figures 2C**, **5A**), and both foliar K concentration and Pmax showed a significant negative relationship with foliar N concentration (**Figure S1**), while the relatively positive response of Pmax of *B. platyphylla* and *P. koraiensis* was accompanied by an increase in their foliar K concentrations (**Figure 2C**), suggesting that the application of N only resulted in K limitation in *F. mandshurica* leaves. We also found that the decrease in foliar K concentration of *F. mandshurica* was accompanied by a decrease in foliar P concentration (**Figure 2B**), which may be caused by the decrease of cation concentrations in plant tissues and the consequent imbalance of other ions (Esteban et al., 2016). Our evidence of K concentrations is based on the fact that the retention of NH_3_ or NH_4_^+^ by leaves can also lead to the leaching of cations from plant tissues and a decrease in cation concentrations. This is due to the exchange of NH_4_^+^ with cations in leaves and the possible competition between NH_4_^+^ (protonated NH_3_ in the apoplast) and cations in leaves, particularly K^+^, in their function to maintain electroneutrality (Van der Eerden et al., 1992; Stachurski and Zimka, 2002; Mori et al., 2019; Turpault et al., 2021). Therefore, according to our findings, our Pmax results can also be explained by the sensitivity of tree species to NH_4_^+^ in the traditional view. Due to the higher NH_4_^+^ sensitivity of *F. mandshurica*, the deposited NH_4_^+^ may exceed the tolerance threshold of *F. mandshurica* and led to P and K limitation in leaves, so the photosynthetic capacity was reduced. For *B. platyphylla* and *P. koraiensis*, because of their lower NH_4_^+^ sensitivity, they may have avoided or alleviated the nutrient limitation in the leaves under NH_x_ deposition, so the deposited NH_4_^+^ was invested into the photosynthetic system or stored in the organic N pool.

In addition, there is also evidence that NO_3_^-^ signaling and reduction in plant tissues are important factors in the alleviation of NH_4_^+^ stress (Hachiya et al., 2012; Esteban et al., 2016). Therefore, in our results (**Figure 3A**), the continuous increase in NR activities in leaves of the three species may be a mechanism by which they alleviate the possible stress caused by deposited NH_3_/NH_4_^+^. The increase in NR activity was much lower of *B. platyphylla* than that of *F. mandshurica* and *P. koraiensis*, suggesting that the stimulation of *B. platyphylla* leaves by deposited NH_3_/NH_4_^+^ was probably the lowest and may be one of the reasons for the relatively most positive response of Pmax of *B. platyphylla*. Combined with the above discussion, we propose that *B. platyphylla* and *P. koraiensis* may better acclimate to canopy NH_x_ deposition than *F. mandshurica*.

It is noteworthy that the foliar K concentration of *B. platyphylla* and *P. koraiensis* decreased significantly under the 0D+100W treatment compared with the control (**Figure 2C**), which may be attributed to the above canopy exchange of cations. However, we did not find the decrease in their physiological function, this may be due to the low NH_4_^+^stress caused by the lower NH_4_^+^ retention in leaves, and the lagged response of the leaves to additional NH_4_^+^ entering the soil through the canopy (Wang et al., 2021). This result suggests that the foliar K concentration was reduced before NH_4_^+^ exceeded the threshold. This was inconsistent with the possible positive relationship between photosynthesis and leaf K status under the increase in *R*_Dry/Wet_ in this study. Therefore, we expect that the acclimation of plants to dry deposition and wet deposition may be completely different. However, it merits a further study to track the effects of long-term alternating dry and wet N deposition on function and cation status of leaves in the future, before a clear conclusion can be reached. Given that studies on the response mechanism of plants to retained N by leaves and plant sensitivity/tolerance to retained N are still limited, the acclimation mechanism of plants under dry and wet N deposition is still an open question.

## 5 Conclusion

Our results show that, relative to wet deposition, dry deposition of N would lead to higher foliar N concentrations and induce a greater response of photosynthesis in plant canopy leaves. Dry deposition is relatively more important on leaf physiological functions in alternating deposition, ignoring alternating processes of dry and wet N deposition would result in a biased assessment of the response of forest canopy to atmospheric N deposition. *B. platyphylla* and *P. koraiensis* may better acclimate to canopy NH_x_ deposition than *F. mandshurica*. Nevertheless, we found that dry and wet deposition had different effects on leaf K status, so we expect that the acclimation of plants to dry deposition and wet deposition may be completely different. Further long-term studies are needed to improve our understanding of how the structure and physiology of canopy leaves respond to alternating dry and wet N deposition under the realistic condition, and acclimation mechanism of plants to dry-wet alternating deposition of N. They will help us to better understand and project the response of forest ecosystems to atmospheric N deposition in the future.

## Data availability statement

The raw data supporting the conclusions of this article will be made available by the authors, without undue reservation.

## Author contributions

XW and CW designed the experiment. CW and XC built the field facility and simulated nitrogen deposition in the chamber. CW and SZ conducted laboratory experiments. CW analyzed the data and wrote the manuscript with the help of XW. All authors contributed to the article and approved the submitted version.
