# Supplementary material for: Alternating processes of dry and wet nitrogen deposition have different effects on the function of canopy leaves: Implications for leaf photosynthesis
Source: Front Plant Sci. 2023 Jan 9;13:1105075. doi: 10.3389/fpls.2022.1105075 (PMC9868767; doi:10.3389/fpls.2022.1105075)

***Supplementary Material***

The following Supporting Information is available for this article:

**Table S1** Leaf anatomical traits of three species affected by different nitrogen application treatments

**Figure S1** Pearson correlation analysis of leaf traits of three species.

**Figure S2** Leaf cross-section structure of three species affected by different nitrogen application treatments

**Table S1** Leaf anatomical traits of three species affected by six canopy nitrogen (N) application treatments for 32 days.

|  | Treatment |  |  |  |  |  |
| --- | --- | --- | --- | --- | --- | --- |
| Species/morphological trait | Control | 0D+100W | 25D+100W | 50D+50W | 75D+25W | 100D+0W |
| *Betula platyphylla* |  |  |  |  |  |  |
| Leaf area (cm^2^) | 28.53 ± 0.84 | 31.54 ± 0.79 | 31.45 ± 2.03 | 30.00 ± 1.16 | 30.42 ± 1.32 | 30.73 ± 1.70 |
| SLA(cm^2^ g^-1^) | 344.67 ± 22.32 | 311.99 ± 14.22 | 343.93 ± 15.65 | 329.39 ± 22.32 | 328.79 ± 12.50 | 327.46 ± 25.36 |
| LMA(mg cm^-2^) | 11.06 ± 0.21 | 11.42 ± 0.32 | 10.41 ± 0.58 | 11.25 ± 0.67 | 11.04 ± 0.26 | 10.68 ± 0.51 |
| *Fraxinus mandshurica* |  |  |  |  |  |  |
| Leaf area (cm^2^) | 11.95 ± 0.78 | 9.46 ± 1.44 | 11.00 ± 0.83 | 10.28 ± 0.88 | 9.53 ± 1.35 | 9.19 ± 1.15 |
| SLA(cm^2^ g^-1^) | 200.13 ± 15.70 | 201.01 ± 9.38 | 203.81 ± 11.68 | 196.02 ± 3.70 | 199.22 ± 19.03 | 208.34 ± 15.47 |
| LMA(mg cm^-2^) | 17.08 ± 1.09 | 16.26 ± 1.59 | 16.75 ± 0.56 | 17.55 ± 0.27 | 17.39 ± 2.10 | 17.54 ± 0.58 |
| *Pinus koraiensis* |  |  |  |  |  |  |
| Leaf area (cm^2^) | 0.67 ± 0.06 | 0.66 ± 0.02 | 0.72 ± 0.03 | 0.74 ± 0.05 | 0.65 ± 0.03 | 0.70 ± 0.05 |
| SLA(cm^2^ g^-1^) | 71.38 ± 6.09 | 71.76 ± 4.04 | 66.37 ± 1.87 | 65.26 ± 5.52 | 64.75 ± 4.12 | 69.46 ± 2.52 |
| LMA(mg cm^-2^) | 42.59 ± 2.20 | 40.53 ± 2.02 | 43.48 ± 2.82 | 44.81 ± 2.48 | 43.91 ± 0.99 | 40.26 ± 2.63 |

SLA, specific leaf area; LMA, fresh leaf mass per area. N application treatments included a control (no application of N) and five types of N deposition with dry to wet ratios of 0:100 (0D+100W), 25:75 (25D+75W), 50:50 (50D+50W), 75:25 (75D+25W) and 100:0 (100D+0W), respectively. Values are mean ± 1 SE (*n* = 5).

**Figure S1** N, foliar nitrogen concentration; Pmax, leaf light-saturated net photosynthesis rate; Chla+b, chlorophyll concentration; Protein, foliar soluble protein concentration; K, foliar potassium concentration; P, foliar phosphorus concentration; NR, foliar nitrate reductase activity; GS foliar glutamine synthetase activity; Fd-GOGAT, foliar glutamate synthase activity; LA, leaf area; SLA, specific leaf area. Pearson correlation analysis of leaf traits of the three species. Red represents positive relationship, and blue represents negative relationship. The value on the right indicates the magnitude of the correlation coefficient. The larger the circle, the stronger the correlation.

**
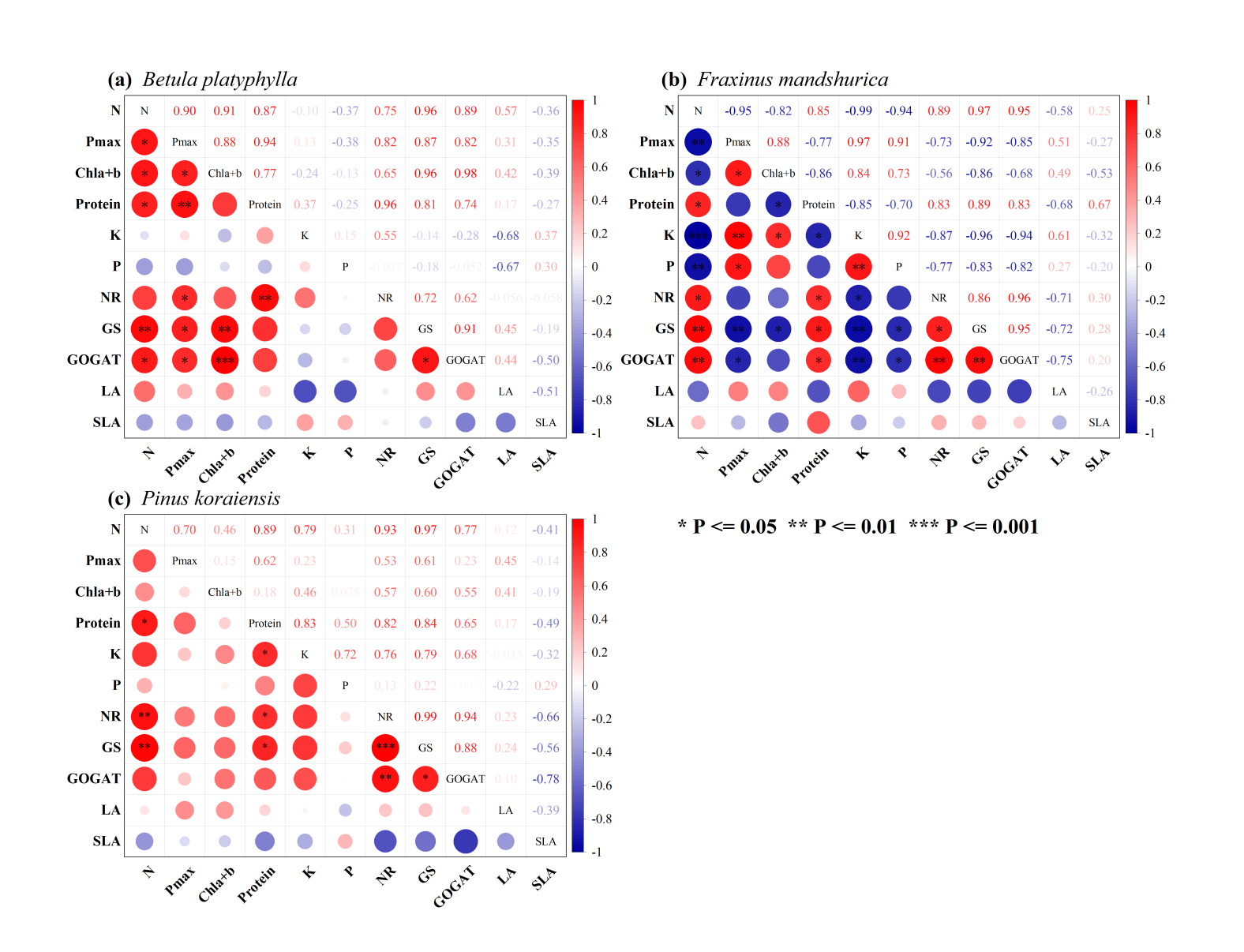
**

**Figure S2** The leaf cross-section structures of three species under six canopy nitrogen (N) application treatments. N application treatments included a control (no application of N) and five types of N deposition with dry to wet ratios of 0:100 (0D+100W), 25:75 (25D+75W), 50:50 (50D+50W), 75:25 (75D+25W) and 100:0 (100D+0W), respectively. Bars, 100 μm.


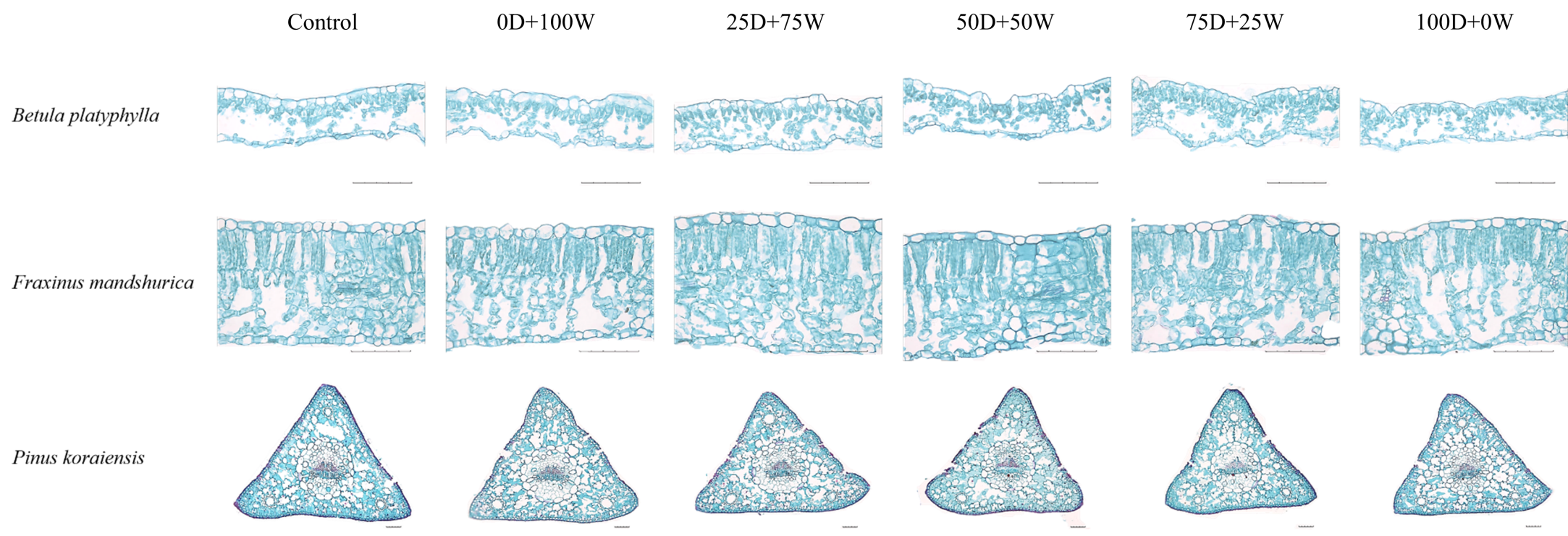

Supplement: Supplementary file 1 [file DataSheet_1.docx]
